# Effects of robot-assisted task-oriented upper limb motor training on neuroplasticity in stroke patients with different degrees of motor dysfunction: A neuroimaging motor evaluation index

**DOI:** 10.3389/fnins.2022.957972

**Published:** 2022-09-16

**Authors:** Hui Xie, Xin Li, Wenhao Huang, Jiahui Yin, Cailing Luo, Zengyong Li, Zulin Dou

**Affiliations:** ^1^Department of Rehabilitation Medicine, The Third Affiliated Hospital of Sun Yat-sen University, Guangzhou, China; ^2^Beijing Key Laboratory of Rehabilitation Technical Aids for Old-Age Disability, National Research Center for Rehabilitation Technical Aids, Beijing, China; ^3^Key Laboratory for Biomechanics and Mechanobiology of Ministry of Education, School of Biological Science and Medical Engineering, Beihang University, Beijing, China

**Keywords:** robot-assisted task-oriented motor training, functional near-infrared spectroscopy, neuroplasticity, cerebral activation, lateralization, functional connectivity, stroke

## Abstract

**Introduction:**

Although robot-assisted task-oriented upper limb (UL) motor training had been shown to be effective for UL functional rehabilitation after stroke, it did not improve UL motor function more than conventional therapy. Due to the lack of evaluation of neurological indicators, it was difficult to confirm the robot treatment parameters and clinical efficacy in a timely manner. This study aimed to explore the changes in neuroplasticity induced by robot-assisted task-oriented UL motor training in different degrees of dysfunction patients and extract neurological evaluation indicators to provide the robot with additional parameter information.

**Materials and methods:**

A total of 33 adult patients with hemiplegic motor impairment after stroke were recruited as participants in this study, and a manual muscle test divided patients into muscle strength 0–1 level (severe group, *n* = 10), 2–3 level (moderate group, *n* = 14), and 4 or above level (mild group, *n* = 9). Tissue concentration of oxyhemoglobin and deoxyhemoglobin oscillations in the bilateral prefrontal cortex, dorsolateral prefrontal cortex (DLPFC), superior frontal cortex (SFC), premotor cortex, primary motor cortex (M1), primary somatosensory cortex (S1), and occipital cortex were measured by functional near-infrared spectroscopy (fNIRS) in resting and motor training state. The phase information of a 0.01 −0.08 Hz signal was identified by the wavelet transform method. The wavelet amplitude, lateralization index, and wavelet phase coherence (WPCO) were calculated to describe the frequency-specific cortical changes.

**Results:**

Compared with the resting state, significant increased cortical activation was observed in ipsilesional SFC in the mild group and bilateral SFC in the moderate group during UL motor training. Patients in the mild group demonstrated significantly decreased lateralization of activation in motor training than resting state. Moreover, the WPCO value of motor training between contralesional DLPFC and ipsilesional SFC, bilateral SFC, contralesional, S1, and ipsilesional M1 showed a significant decrease compared with the resting state in the mild group.

**Conclusion:**

Robot-assisted task-oriented UL motor training could modify the neuroplasticity of SFC and contribute to control movements and continuous learning motor regularity for patients. fNIRS could provide a variety of real-time sensitive neural evaluation indicators for the robot, which was beneficial to formulating more reasonable and effective personalized prescriptions during motor training.

## Introduction

A stroke could cause the death of brain cells and consequently the loss of abilities controlled by the interested area of the brain, which was the leading cause of a patient's long-term disability (Morone et al., 2020). Most stroke survivors have acute-stage upper limb (UL) dysfunction and only 10–18% of stroke survivors obtain complete UL functional recovery after 6 months post-stroke (Aprile et al., 2020; Morone et al., 2020). In the remaining patients, UL motor deficits persist with a negative impact on their physical and social activities (Harvey, 2015; Waddell et al., 2016). Therefore, an adequate and timely rehabilitation of UL motor function after stroke for a patient was a fundamental demand, while all the new technologies available should also be utilized.

Robot-assisted task-oriented UL motor training had been shown to be effective for UL functional rehabilitation after stroke (Rensink et al., 2009). The previous fMRI neuroimaging had proven that integrating arm training into tasks could induce a broader range of brain activation than usual therapy, such as the visuospatial, visual, and sensory regions, as well as the primary auditory cortex (Wu et al., 2020). However, several studies (Mehrholz et al., 2018; Morone et al., 2020), including the Lancet (Rodgers et al., 2019), had shown that robot-assisted training and advanced therapy did not improve UL motor function more than usual therapy in patients after stroke due to the lack of clear therapy dose studies, such as treatment duration and strength, frequency of sessions, and possible side effects (Morone et al., 2020). Given that the optimal plan of therapy needed for patients after stroke was not exactly known, it was fundamental to identify the parameters of robotic therapy according to an individualized neurorehabilitation program for each specific patient, as well as verify its efficacy using a valid objective assessment.

For task-oriented UL motor training, a single movement could be performed many times always in the same manner, and robots could evaluate UL functions through these objectively physical parameters, such as muscle strength, muscle tension, and joint range of motion (Duret et al., 2019), rather than neural indicators. However, understanding the neuroplastic changes during UL training was crucial in rehabilitating stroke patients, which would directly affect the parameter selection and clinical effectiveness of robot-assisted task-oriented UL motor training (Cramer et al., 2012; Pekna et al., 2012). Functional near-infrared spectroscopy (fNIRS), as a recently developed neuroimaging technology, had the unique advantages of millisecond temporal resolution, 2–3 cm spatial resolution, portability, and low disturbance by movement (Kato et al., 2002; Liu et al., 2015; Chao-Chen et al., 2018), which was suitable for non-invasive assessment to determine the change of neuroplasticity in patients with subcortical and cortical stroke during UL motor training. Current robots needed to be more intelligent and more reliable in clinical practice (Ai et al., 2021). Combining neuroimaging information with machine learning algorithms could enable robots to identify and predict the future rehabilitation direction from neural data, which was conducive to improving the accuracy and effectiveness of robot-assisted UL rehabilitation.

Neuroplasticity referred to the brain's ability to undergo functional and structural changes in response to external or internal stimuli from the environment or organs in the body, and could also be comprehended as an obligatory adaptation in response to each neurobiological process (Pascualleone et al., 2005; Smith, 2013; Patrice et al., 2017). Long-term potentiation (LTP) was the process of neuroplasticity, which could be divided into early and late LTP. Early LTP produced a rapid and short-lasting alteration of neuroplasticity changes and continuously transitioned to late LTP that produces slower and longer-lasting plasticity (Loprinzi, 2019; Bandeira et al., 2021). With neuroimaging technology, the early neuroplasticity process could be evaluated by real-time detection of the specific task, while the late neuroplasticity process needed to be obtained by comparing resting states in a long-term follow-up study. In this study, we hypothesized that patients with different degrees of dysfunction would have a variety of patterns of brain network reorganization during UL rehabilitation training. Therefore, the current study aimed to (1) explore the specific changes of neuroplasticity during rehabilitation induced by robot-assisted task-oriented UL motor training in patients with different degrees of dysfunction, and (2) extract neurological evaluation indicators that could be identified by machine learning, so as to provide the robot with additional neurological parameters information other than physical parameters.

## Materials and methods

### Participants

A total of 33 (*n* = 33) right-handed adult patients with hemiplegic motor impairment after stroke were recruited as participants in this fNIRS study through inpatients of the Third Affiliated Hospital of Sun Yat-sen University, and the patient's clinical baseline characteristics as shown in Table 1. The inclusion criteria were (1) the First stroke, confirmed by cranial CT or MRI. (2) Time of onset between 1 week and 6 months. (3) Presence of mild, moderate, or severe motor dysfunction. (4) Patients without significant cognitive and verbal dysfunctions (MMSE > 21 points). Participants were excluded from the study if they had: (1) Prior history of stroke, traumatic brain injury, or brain tumor. (2) Complicated severe cardiac, pulmonary, hepatic, or renal dysfunction or other serious physical illness. (3) Previous epilepsy and family history of epilepsy. (4) Metals implanted in the body such as pacemakers, metals in the skull, etc. (5) Those with new infarct foci or secondary hemorrhages that have worsened. (6) Those with severe cervical spine lesions including severe cervical canal stenosis and cervical instability. The trial was registered under the Chinese Clinical Trial Register no. ChiCTR2100054527 (Registered 19 December 2021). Ethical approval was granted by the Ethics Committee of the Third Affiliated Hospital, Sun Yat-sen University.

**Table 1:** Characteristics of participants with UL motor dysfunction.

**Patients**	**Sex**	**Age**	**Etiology**	**Hemiplegia side**	**Site of lesion**	**Time (month)**	**MMT**
Pt 1	Male	54	Infarction	Left	Frontotemporal-parietal	0.9	0
Pt 2	Male	45	Hemorrhage	Right	External capsule	2.77	1
Pt 3	Female	78	Infarction	Left	Internal capsule	0.4	1
Pt 4	Female	80	Infarction	Right	Pons	2.27	4
Pt 5	Male	84	Infarction	Left	Cerebellum	1.13	2
Pt 6	Female	43	Infarction	Right	Basal ganglia	0.5	4
Pt 7	Male	62	Infarction	Right	Temporal lobe	0.67	5
Pt 8	Male	62	Infarction	Right	Frontoparietal	0.9	4
Pt 9	Male	70	Infarction	Left	Basal ganglia	1.33	1
Pt 10	Male	58	Hemorrhage	Right	Basal ganglia	1.07	2
Pt 11	Male	33	Infarction	Right	Middle cerebral artery	2.43	2
Pt 12	Male	50	Infarction	Left	Frontoparietal lobe	2.3	5
Pt 13	Male	64	Infarction	Left	Vertebral artery	3.97	1
Pt 14	Female	64	Infarction	Right	Pons	1.63	2
Pt 15	Male	68	Infarction	Right	Basal ganglia	1.43	2
Pt 16	Male	45	Infarction	Right	Basal ganglia	4.9	2
Pt 17	Male	43	Hemorrhage	Left	Pons	1.43	4
Pt 18	Female	62	Hemorrhage	Left	Parietal lobe	4.01	1
Pt 19	Male	89	Infarction	Left	Pons	2.5	2
Pt 20	Male	32	Hemorrhage	Left	Parietal lobe	2.53	4
Pt 21	Male	65	Infarction	Left	Basal ganglia	2.37	2
Pt 22	Female	63	Infarction	Right	Pons	0.7	4
Pt 23	Male	55	Hemorrhage	Left	Basal ganglia	0.6	0
Pt 24	Male	60	Hemorrhage	Left	External capsule	0.67	2
Pt 25	Male	50	Infarction	Right	Basal ganglia	0.43	3
Pt 26	Female	73	Infarction	Right	Corona radiata	1.2	1
Pt 27	Male	44	Infarction	Right	Basal ganglia	1.27	2
Pt 28	Male	50	Infarction	Right	Vertebral artery	0.4	0
Pt 29	Male	42	Hemorrhage	Left	Basal ganglia	0.27	2
Pt 30	Female	55	Infarction	Right	Basal ganglia	5.8	2
Pt 31	Male	54	Hemorrhage	Left	Basal ganglia	2.07	3
Pt 32	Female	72	Infarction	Right	Internal capsule	2.33	2
Pt 33	Male	35	Hemorrhage	Left	Basal ganglia	4.8	1

### Procedures

**Figure 1:**
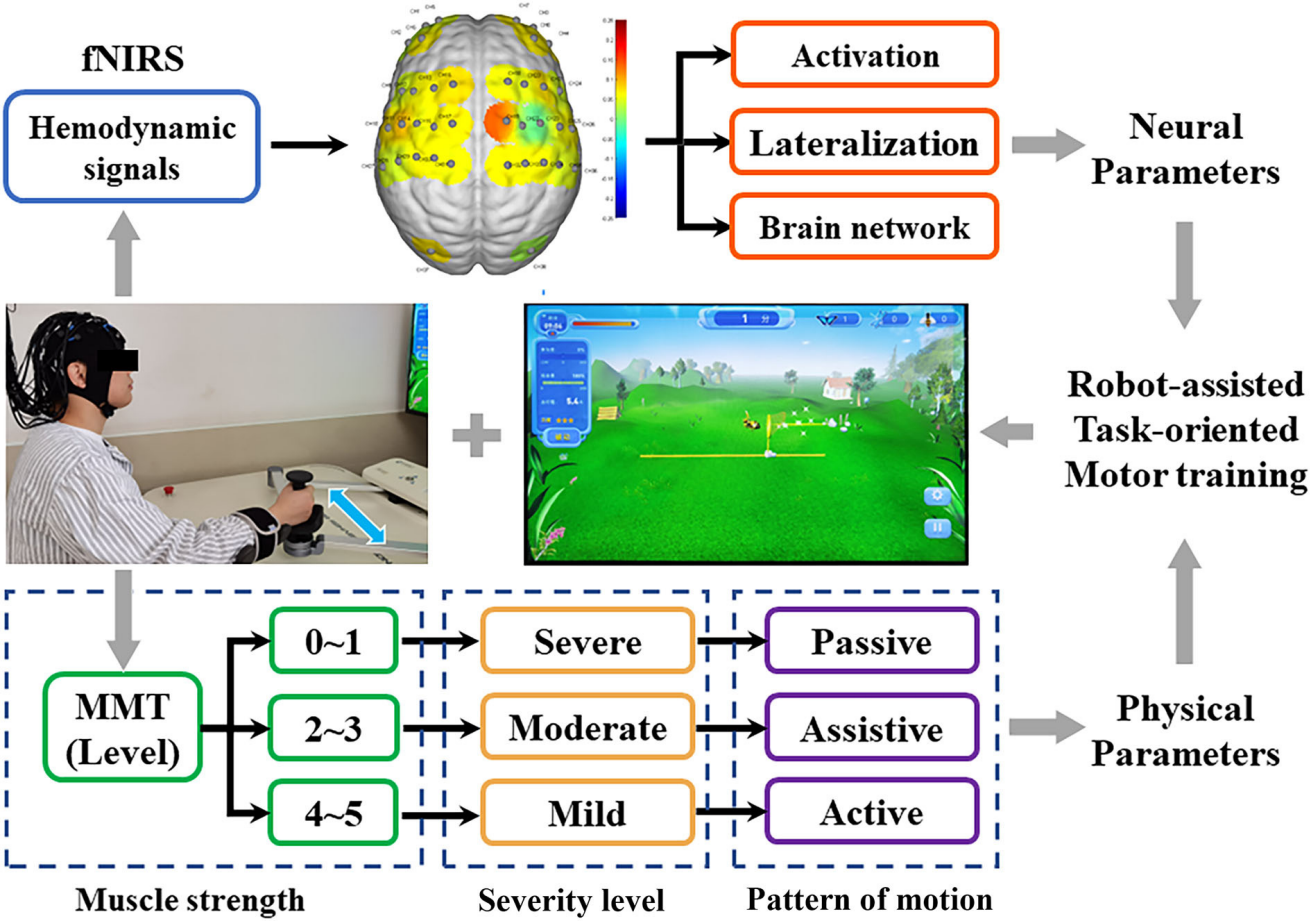
Experimental protocol. The fNIRS technique was used to detect the real-time hemodynamic signal of patients with three different degrees of UL dysfunction during motor training. Activation, lateralization, and brain network as neural parameters were used to evaluate fNIRS signals.

All patients received a robot-assisted task-oriented UL motor training (ArmGuider, ZD Medtech Co., Ltd., China), and the robot automatically adjusted the resistance or assist parameters according to the manual muscle test (MMT) results of the patient's hemiplegic UL post-stroke. In detail, muscle strength at 0–1 level corresponds to the passive motor (severe group, *n* = 10), at level 2–3 correspond to the assistive motor (moderate group, *n* = 14), and at level 4 or above corresponds to activities including resistance motor (mild group, *n* = 9). The patient's distal hand was fixed on a robotic arm, and training directions included horizontal shoulder adduction and abduction, elbow flexion, and extension. Each stroke patient received 20 min per day, 5 days per week of robot-assisted task-oriented UL training. The experiment was conducted by a professional therapist in a silent treatment room. Before the experiment, all participants were required to sit for 5–10 min to eliminate existing hemodynamic reactions induced by their activity. Because FNIRS signal should include at least five low-frequency periods (0.01 Hz) to ensure the effectiveness of phase correlation analysis (Bandrivskyy et al., 2004), patients were asked to complete a 10-min resting state and a 10-min motor training state in a sitting position. In the resting state, the patient was asked to relax the brain and avoid random movements and speech. In the motor training state, patients were seated in front of the training table, and their hands and forearms were fixed on the movable arm of the robot. Then, the maximum range of motion of the hemiplegia UL was set. Patients were asked to complete a dynamic task of catching butterflies on a screen through the robot's movable arm. The fNIRS was implemented continuously throughout the experiment and the set-up is shown in Figure 1.

### Functional near–Infrared spectroscopy measurement

**Figure 2:**
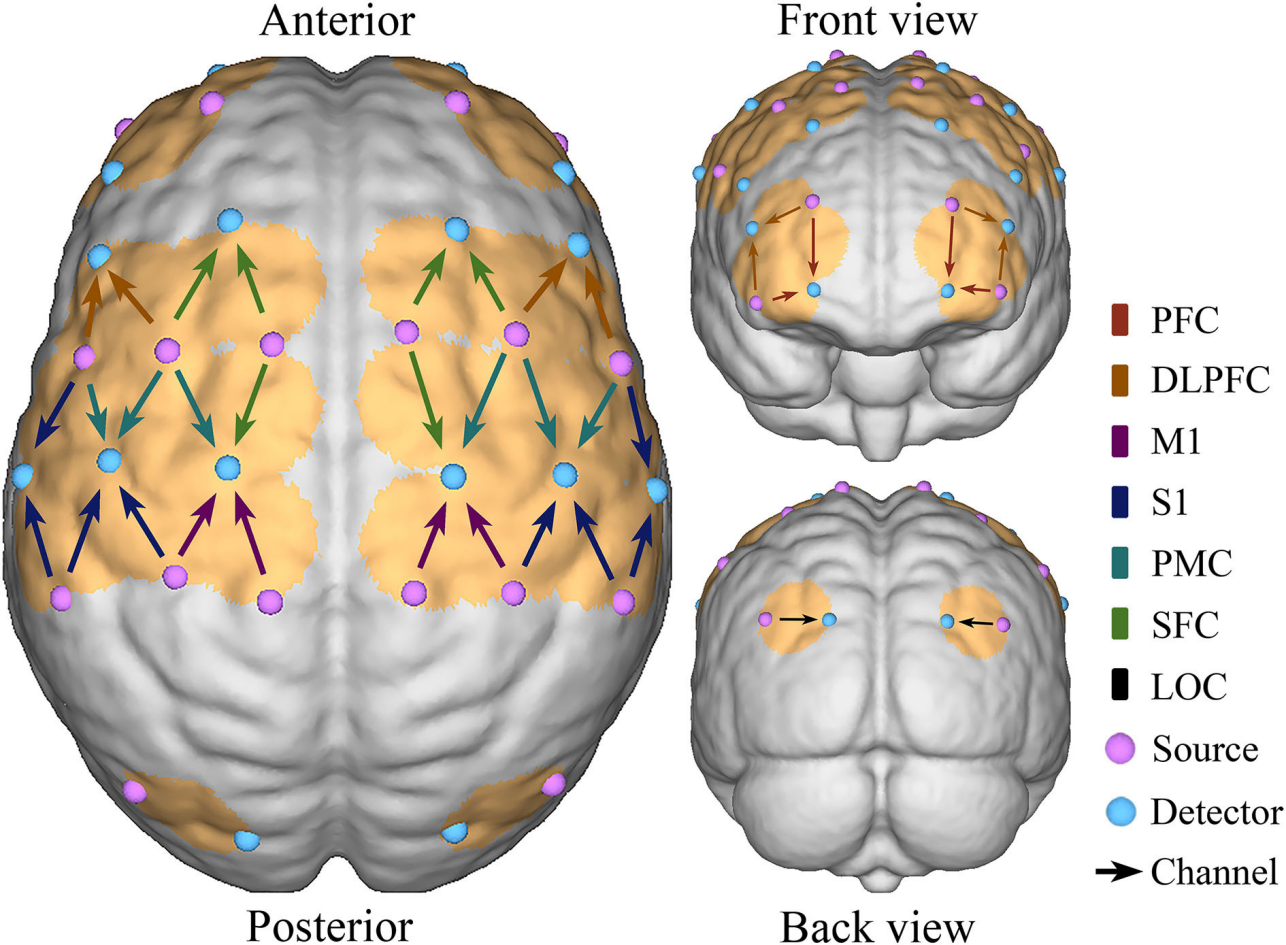
Schematic diagram of the fNIRS. Configuration of 18 source probes, 16 detector probes, and 38 measurement channels.

A multi-channel tissue oxygenation monitor with continuous wave (NirSmart, Danyang Huichuang Medical Equipment Co., Ltd., China) with wavelengths of 740 and 850 nm (Nieuwhof et al., 2016) was used in fNIRS measurements. Each sensor of the instrument consisted of a light emitting diode and a detector optode with a distance of 30 mm and the sampling rate was 10 Hz. The calibration function of the instrument and the corresponding template was used to ascertain the channels to fill exactly in correspondence of the 10/10 electrode positions according to different head sizes. A total of 38 measurement channels, including 18 light source probes and 16 detector probes, were symmetrically positioned over the regions of the ipsilesional and contralesional prefrontal cortex (IPFC/CPFC), ipsilesional and contralesional dorsolateral prefrontal cortex (IDLPFC/CDLPFC), ipsilesional and contralesional superior frontal cortex (ISFC/CSFC), ipsilesional and contralesional premotor cortex (IPMC/CPMC), ipsilesional and contralesional primary motor cortex (IM1/CM1), ipsilesional and contralesional primary somatosensory cortex (IS1/CS1), and ipsilesional and contralesional occipital cortex (IOC/COC), as shown as Figure 2.

### Data pre-processing

The pre-processing method of fNIRS data had been elaborated in our previous studies (Tan et al., 2015, 2016; Xu et al., 2017; Xie et al., 2019). The absorbance signals recorded by fNIRS were first bandpass filtered at 0.0095–2 Hz (zero-phase, six-order Butterworth filter) to reduce the uncorrelated noise components and low-frequency baseline drift. Then principal component analysis (PCA) and independent component analysis (ICA) were performed on delta oxygenated hemoglobin (O_2_Hb) and deoxygenated hemoglobin (HHb) signals of each channel to identify and eliminate the components that might be related to noise and artifacts, including cardiac pulsations, respiratory signals, and blood pressure changes (Zhang et al., 2010; Santosa et al., 2013). According to the criteria that the associated time course should have a significant 0.01–0.08 Hz frequency spectrum, the components of interest were visually identified and retained, indicating the functional hemodynamic response in the brain. Finally, a moving average filter was used to eliminate the obvious abnormal points in the signal, and the time window of the moving average filter was 3 s. The artifact portion was removed by cubic spline interpolation.

### Wavelet transform and amplitude

Continuous wavelet transformation could project time series from the time domain to the frequency domain and enable us to continuously derive the frequency content in time by adjusting the length of wavelet windows. The specific frequency interval distinguished by the wavelet transform had different physiological sources, and 0.01–0.08 Hz indicated the neural activity hemodynamic response in the spontaneous cerebral oxygen signal (Lu et al., 2007). The results of the wavelet transform were averaged over the time domain to obtain the wavelet amplitude (WA) of each delta O_2_Hb and HHb signal at each time and frequency, which reflects the magnitude of the fluctuation of the original signal at a certain frequency. WA of the delta O_2_Hb and HHb signal represents the changes in regional cerebral blood flow with the activity of the cerebral cortex during different conditions. Functional hyperemia or neurovascular coupling could increase regional cerebral blood flow by activating local neurons to match the needs of blood and nutrients of local brain cells in the task state (Willie et al., 2014). Thus, WA was characterized by the intensity or activation of the cerebral cortex.

### Lateralization index

The lateralization index (LI) could evaluate the degree of hemispheric activation balance during robot-assisted task-oriented UL motor training. LI for a given contralesional (C) and ipsilesional (I) hemispheric activation was calculated by the sum of the WA values, and the definition was as follows (Calautti and Baron, 2003):


$$LI_{region}=\frac{\left(\sum WA_{C-region}-\sum WA_{I-region}\right)}{\left(\sum WA_{C-region}+\sum WA_{I-region}\right)}$$

The value of LI ranges from 1 (contralesional activation only) to −1 (ipsilesional activation only).

### Wavelet phase coherence

The functional connectivity was calculated using wavelet phase coherence (WPCO), which was a method of using the phase information of the signal to evaluate the correlation between two signals. The WPCO value was between 0 and 1, and the value quantitatively represents the instantaneous phase of the two signals at a consistent degree throughout the continuous process of the time series to identify possible connectivity (Bernjak et al., 2012). The high WPCO value indicates that an agreement between the two cortical regions exists, otherwise it indicates weaker relationships between the two existing delta signals (Han et al., 2014).

To identify significant coherence, the amplitude–adaptive Fourier transform method was applied to perform the WPCO test. A total of 50 surrogate signals with the same mean, variance, and autocorrelation functions as the original signal but without any phase correlation were produced. The phase coherence level of the original signal was verified by calculating the substitute signals. When the WPCO value of the original signal was higher than two standard deviations above the mean of the surrogate signal, the connectivity in the frequency interval was considered significant (Tachtsidis et al., 2004).

### Statistical analysis

Data were analyzed using the Kolmogorov–Smirnov test and Levene test to ensure that the assumptions of normality and homogeneity of variance required to analyze the parameters were satisfied. One-way ANOVA was used to evaluate the significant differences in region-wise WA, LI, and WPCO values among the intra-group comparisons, including resting state vs. UL motor training state in the severe, moderate, and mild groups, the adjusted *p*-value threshold was set at *p* < 0.0167 (0.05/3).

## Results

### Differences in cerebral activation

**Figure 3:**
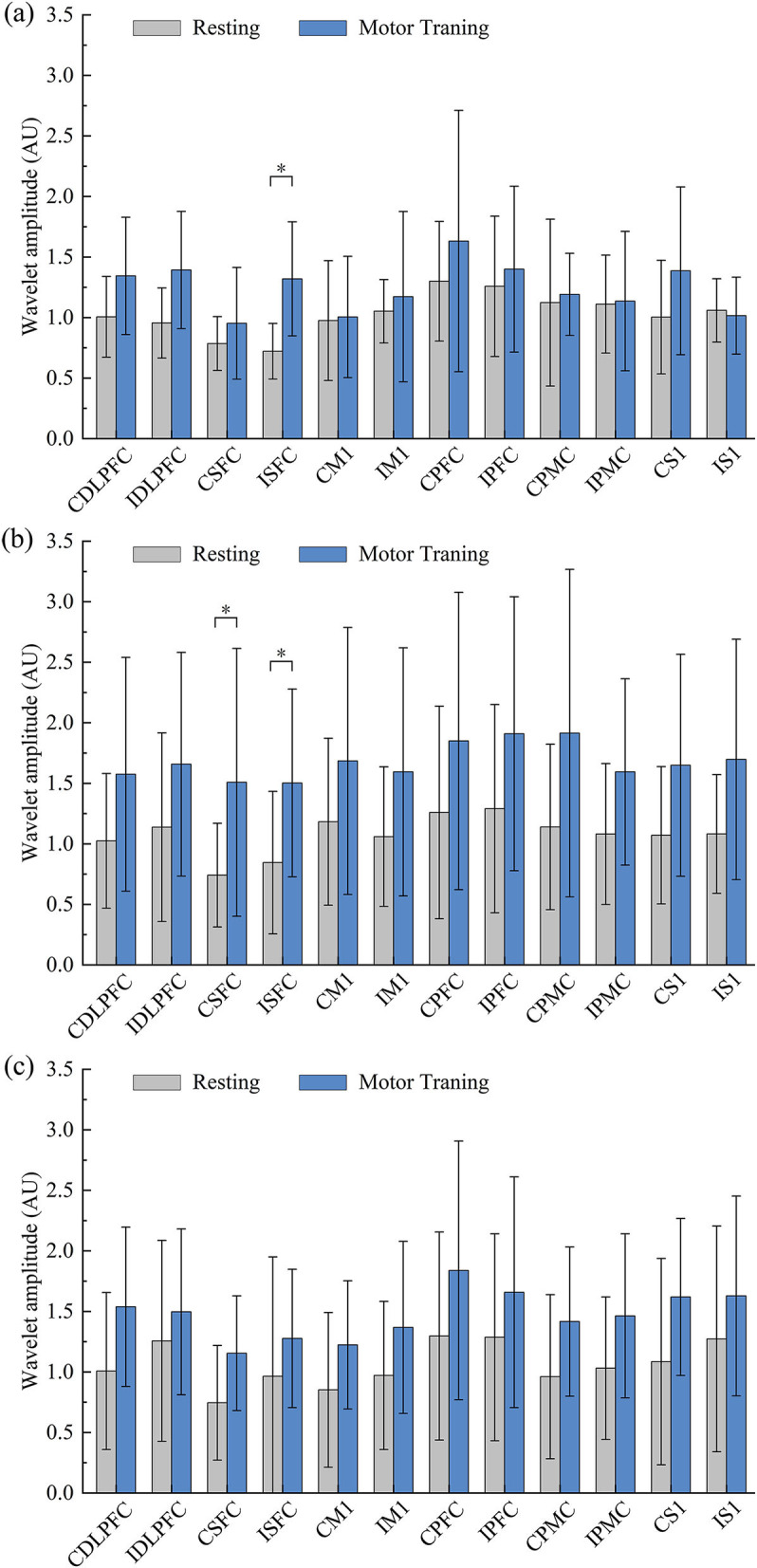
Comparative results for WA values between resting and training states in mild **(a)**, moderate **(b)**, and severe **(c)** groups (*p < 0.05).

Compared with the resting state, the WA changes in the robot-assisted task-oriented UL training of mild (a), moderate (b), and severe (c) groups are shown in Figure 3. We found that the WA values of all regions in the three groups showed different degrees of change. In detail, the WA value of ISFC (*F* = 9.092, *p* = 0.011) showed a significant increase in the mild group in motor training compared to the resting state. In the moderate group, the region's activation was generally higher than in the other groups, and a significant increase of WA was observed in ISFC (*F* = 5.938, *p* = 0.023) and CSFC (*F* = 5.425, *p* = 0.029). However, although the motor training in the severe group could induce a wide range of activation increases, there were no significant changes compared with the resting state.

### Differences in brain lateralization

**Figure 4:**
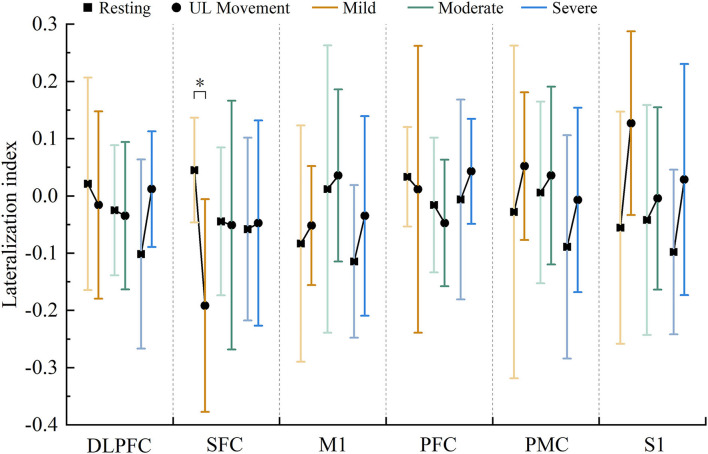
Changes in the LI value in each region under resting state and motor training state in patients with mild (red), moderate (green), and severe (blue) motor dysfunction (* *p* < 0.05).

Compared with the resting state, the results of the LI value showed a significant decrease in SFC (*F* = 9.122, *p* = 0.001) in the motor training state in the mild group. However, there was no evidence that the LI values showed significant differences in robot-assisted task-oriented UL motor training compared with resting in moderate and severe groups, as shown in Figure 4. Moreover, the result observed that activation in M1, PMC, and S1 was lateralized to the contralesional hemispheric. In contrast, the LI of DLPFC, PFC, and SFC showed a decrease in mild and moderate groups, while showing an increase in the severe group.

### Differences in brain network connectivity

**Figure 5:**
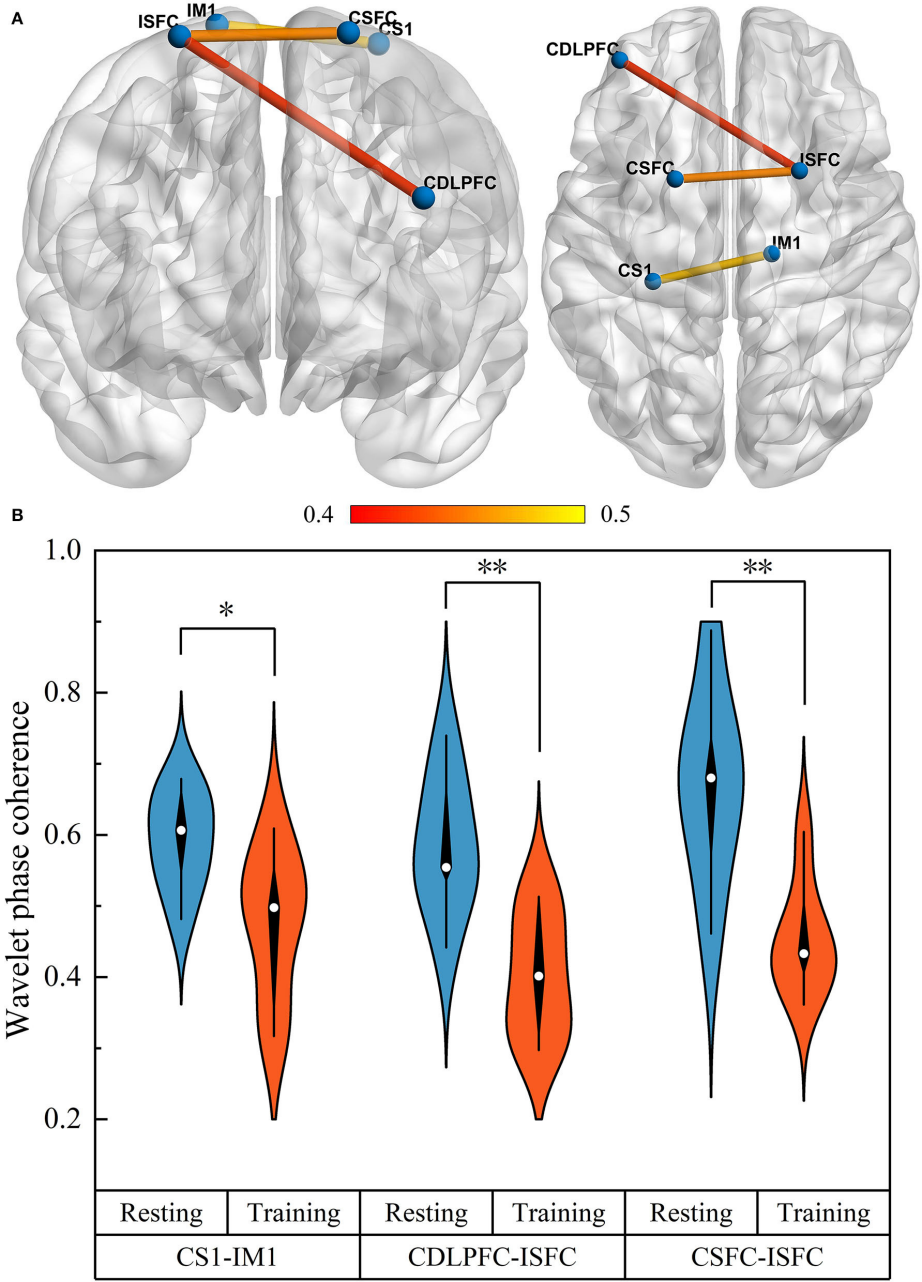
The functional connectivity visual map **(A)**. The connectivity line indicates a significant WPCO value between the two regions. Line color indicates the connectivity intensity, and the brighter color represents higher strength. The result of significant changes of WPCO values in motor training compared with resting state **(B)** (*p < 0.05; ** *p* < 0.01).

We examined changes in WPCO values in motor training compared to the resting state in three groups. A significant difference in the WPCO values related to the UL motor training was found in the mild and moderate groups, and the significant change was shown in a visual connectivity map, as shown in Figure 5A. In detail, the functional connectivity result showed that the WPCO value of motor training between CDLPFC and ISFC (*F* = 9.572, *p* = 0.009), CSFC and ISFC (*F* = 13.694, *p* = 0.003), and CS1 and IM1 (*F* = 6.167, *p* = 0.029) were significantly lower than resting state, as shown in Figure 5B, and there was no significant difference in the WPCO value was found between resting and motor training in the severe group.

## Discussion

This study mainly observed the changes in neuroplasticity in patients after stroke with different degrees of UL motor dysfunction during the robot-assisted task-oriented UL motor training. The main finding was that robot-assisted task-oriented UL motor training could significantly increase the nerve activation response of SFC in mild and moderate patients. In addition, the lateralization and brain function network related to ISFC had significantly changed in patients with mild motor dysfunction. However, there was no evidence that robot-assisted task-oriented UL motor training could significantly change the neuroplasticity of patients with severe motor dysfunction after stroke.

Previous studies had shown an association between improved clinical efficacy and brain activation. A recent quantitative meta-analysis found that better motor performance was associated with the likelihood of greater activation in the ipsilesional hemisphere (Hubbard et al., 2015). In detail, the finding that increased brain activation in motor-related and attentional regions was associated with recovery of UL function in those that received motor training during stroke rehabilitation was clinically relevant. We contended that the robot-assisted task-oriented UL motor training had the potential to increase opportunity for attentive and task-specific of motor function in patients with partial motor ability. This process would be expected to include brain regions involved with motor learning and attention (Hubbard et al., 2015), such as SFC, which is consistent with our findings and with learning-dependent plasticity.

Evidence increasingly identified that the supplementary motor area, located in the middle and posterior SFC, was important to UL recovery in the first 6 months post-stroke (Kokotilo et al., 2010; Carey et al., 2011). The current study's findings supported the theory that stroke recovery might be associated with the recruitment of spared motor and attention regions (Barch et al., 2000; Kokotilo et al., 2009; Buma et al., 2010). There had been wide discussion on the role of contralesional hemispheric compensation in the functional rehabilitation of the paralyzed UL. The ipsilesional pathway had great potential for controlling both hands using the contralesional hemisphere, which could account for 10–20% of all corticospinal projections (Riecker et al., 2010; Hua et al., 2016). Therefore, enhanced contralesional hemisphere activation might successfully compensate for motor control for patients after stroke. In addition, we realized that other regions of the brain might also be making a functional contribution, but did not show a significance in this study. Although robot-assisted task-oriented UL motor training could affect multiple brain regions in patients with different degrees of motor dysfunction, the magnitude of the neural activation changes seemed to depend on the intensity of motor training.

One of the primary interests of this study was to assess the hemispheric activation balance. The different activation intensities of regions determine the distribution of brain resources, and the lateralized activation pattern of the cortex was a known factor determining the degree of motor function recovery in patients after stroke. In this study, the lateralization of most brain regions was consistent in patients with different degrees of motor dysfunction. It was worth noting that the function of ipsilesional hemispheric SFC was dominant during motor training in mild patients, which might be an important sign of UL functional rehabilitation. In addition, IPFC and IDLPFC exhibited an increasing trend of laterality in mild and moderate patients during UL motor recovery. Although PFC and DLPFC were not considered primary motor regions, the activation of the IPFC and IDLPFC might be beneficial to enhance the management of the cognitive load required for motor performance (Buckner et al., 2008; Hussar and Pasternak, 2013; Nee and D'esposito, 2016). However, the laterality trend of these regions was opposite in patients with severe motor dysfunction, which might reflect the differences in the mechanism of brain network reorganization between different degrees of dysfunction patients. Neuroimaging studies had shown that the grasp of motor regularity in hemiplegia patients depended on the activation degree of the CDLPFC region (Calautti et al., 2010). Robot-assisted task-oriented training could force severely affected patients to invest extra conscious attention to continue learning and follow the cues, which was thought to be an effective way for them to gain insight into their own behavior and regain a reactive strategy to achieve optimal task performance. Therefore, adequate CPFC and CDLPFC engagement appeared essential for stroke survivors to recover UL motor function. Although the existing evidence in this study did not show that robot-assisted task-oriented UL motor training had a significant effect on the LI change in moderate and severe patients, this trend was still worthy of attention.

The results of the brain network showed significantly decreased functional connectivity between the ISFC-CDLPFC and ISFC -CSFC in mild dysfunction patients, which reflected the emergence of a dyssynchrony phenomenon in interhemispheric corticocortical connections involved in cognitive-motor function during dynamic brain network reorganization. On the one hand, as mentioned above, the contralesional frontal region was involved in the continuous and repetitive learning of motor regularity. On the other hand, the previous study had been demonstrated that increasing task difficulty results in greater ipsilesional motor-related activation (Horenstein et al., 2009). Taken together, it seemed reasonable to assume that robot-assisted task-oriented training plays a key role in activating motor memory to control skilled movements in patients with preserved UL muscle strength, as well as in continuously learning motor regularities in patients with severe motor dysfunction.

In addition, we found that the evidence of inducing significant differences in neuroplasticity was diminishing with the severity of the dysfunction. Therefore, it seemed to be the correct selection to transition from relying solely on external assistance to a more challenging UL motor training by reducing assistance or even increasing resistance in a timely manner according to the changes in the patient's muscle strength during the rehabilitation of UL function. Although the passive motor was generally addressed to decrease the muscular tension and increase the active range of motion in the early rehabilitation stage (Pan et al., 2012), it was more important to highlight changes in neuroplasticity in the rehabilitation of patients after stroke, which determined the ability of the nervous system to alter its structure and function to adapt to changes in the internal and external environment. There was increasing agreement that the number of neurons and the strength of the neural networks involved in a task were directly related to the intensity and amount of practice that had been done (Koes et al., 2001; Lindberg et al., 2004). Therefore, the robot-assisted task-oriented UL training should be of such an intensity to drive structural and functional changes in the central nervous system. It is obvious that our UL motor training did not have sufficient intensity to improve large-scale neuroplasticity in this study, especially for patients with severe UL motor dysfunction. One of the most obvious advantages of robotic therapy over conventional therapy was the reduction in personnel resources for a similar amount of practice time (Bustamante Valles et al., 2016; Jakob et al., 2018), which means that increasing the intensity of training within the patient's tolerance would be easily achievable in the future. Even though there was consensus that patients with stroke should receive early and more intensive training, uncertainty remains about how much additional intervention was required to ensure that neuroplasticity could be induced. Further investigation with neuroimaging tools is required.

Our study has several limitations. First, short channels were not used in this study. Although we currently employ an effective pretreatment method combining PCA and ICA to separate out scalp blood pressure, skin blood interference, and non-evoked hemodynamic components and remove unnecessary sources from the hemodynamic response (Scholkmann et al., 2014; Pfeifer et al., 2017), the short channel should be used as a standardized step in future studies. Second, there was no classification of cortical and subcortical stroke. The lesion locations of cortical stroke mainly included frontal, parietal and temporal lobes, while subcortical stroke mainly included corona radiata and basal ganglia. Patients with different lesions might have different sensitivity to UL rehabilitation, and it is valuable to further analyze the influence of different lesion locations on neuroplasticity during motor training. Third, this study did not focus on the effects of neuroplasticity of long-term UL training. In the future, more participants need to be recruited and followed up in order to compare the different changes in the clinical efficacy of robot-assisted task-oriented UL motor training based on neuroimaging motor evaluation indicators.

In conclusion, this study used fNIRS to examine specific changes in cortical reorganization in patients with different degrees of motor dysfunction during training intervention. Our findings demonstrate that the robot-assisted task-oriented UL motor training could modify the neuroplasticity of SFC in patients with mild and moderate motor dysfunction. During training, brain network connections in patients with mild motor dysfunction were altered, which could improve the patient's ability to control movement. The LI of patients with severe motor dysfunction had a tendency to lateralize the contralesional region, which might indicate relearning of motor regularity. In addition, it was also necessary to continuously reduce external assistance in a timely manner with the progress of rehabilitation training and increase the intensity of training within the patient's tolerance. These findings suggested that fNIRS could provide a variety of real-time sensitive neural evaluation indicators for UL training, which would be beneficial for robots to intelligently formulate accurate and effective personalized motor training prescriptions based on the obtained physical parameters and combined with neural parameters.

## Data availability statement

The raw data supporting the conclusions of this article will be made available by the authors, without undue reservation.

## Ethics statement

The studies involving human participants were reviewed and approved by Human Ethics Committee of the Third Affiliated Hospital of Sun Yat-sen University. The patients/participants provided their written informed consent to participate in this study. Written informed consent was obtained from the individual(s) for the publication of any potentially identifiable images or data included in this article.

## Author contributions

HX, XL, WH, JY, CL, ZD, and ZL worked together to complete the manuscript. ZD and ZL contributed to the conception and design of the study and provided opinions on grammar and rhetoric. HX, XL, WH, and CL carried out the experiments. HX and XL performed the data analyses and wrote the manuscript. HX, XL, and JY provided statistical assistance and support. All authors contributed to manuscript revision, read, and approved the submitted version.

## Funding

This project was supported by the National Key Research and Development Project (Grant No. 2020YFC2004205), the National Natural Science Foundation of China (Grant Nos. 11732015, 61675013, 81672249, and 81972154), the Key Realm R&D Program of Guangzhou (Grant No. 202007030007), and the Fundamental Research Funds for Central Public Welfare Research Institutes (Grant No. 118009001000160001).

## Conflict of interest

The authors declare that the research was conducted in the absence of any commercial or financial relationships that could be construed as a potential conflict of interest.

## Publisher's note

All claims expressed in this article are solely those of the authors and do not necessarily represent those of their affiliated organizations, or those of the publisher, the editors and the reviewers. Any product that may be evaluated in this article, or claim that may be made by its manufacturer, is not guaranteed or endorsed by the publisher.

## References

[B1] Ai Q. Liu Z. Meng W. Liu Q. Xie S. Q. (2021). “Machine learning in robot assisted upper limb rehabilitation: a focused review,” in IEEE Transactions on Cognitive and Developmental Systems. p. 1. 10.1109/TCDS.2021.3098350

[B2] Aprile I. Germanotta M. Cruciani A. Loreti S. Pecchioli C. Cecchi F.. (2020). Upper limb robotic rehabilitation after stroke: a multicenter, randomized clinical trial. J. Neurol. Phys. Ther. 44, 3–14. 10.1097/NPT.0000000000000295 31834217

[B3] Bandeira I. D. Lins-Silva D. H. Barouh J. L. Faria-Guimares D. Lucena R. (2021). Neuroplasticity and non-invasive brain stimulation in the developing brain. Progr. Brain Res. 264, 57–89. 10.1016/bs.pbr.2021.04.003 34167665

[B4] Bandrivskyy A. Bernjak A. Mcclintock P. Stefanovska A. (2004). Wavelet phase coherence analysis: application to skin temperature and blood flow. Cardiov. Eng. Int. J. 4, 89–93. 10.1023/B:CARE.0000025126.63253.43

[B5] Barch D. M. Braver T. S. Sabb F. W. Noll D. C. (2000). Anterior cingulate and the monitoring of response conflict: Evidence from an fMRI study of overt verb generation. J. Cogn. Neurosci. 12, 298–309. 10.1162/089892900562110 10771413

[B6] Bernjak A. Stefanovska A. Mcclintock P. V. E. Owen-Lynch P. J. Clarkson P. B. M. (2012). “Coherence between fluctuations in blood flow and oxygen saturation,” in The Random and Fluctuating World: Celebrating Two Decades of Fluctuation and Noise Letters. p. 345–356. 10.1142/9789811252143_0033

[B7] Buckner R. L. Andrews-Hanna J. R. Schacter D. L. (2008). “The brain's default network - Anatomy, function, and relevance to disease,” in A. Kingstone and M.B. Miller. *Year in Cognitive Neuroscience*. p. 1–38. 10.1196/annals.1440.011 18400922

[B8] Buma F. E. Lindeman E. Ramsey N. F. Kwakkel G. (2010). Functional neuroimaging studies of early upper limb recovery after stroke: a systematic review of the literature. Neurorehabil. Neural Repair 24, 589–608. 10.1177/1545968310364058 20439501

[B9] Bustamante Valles K. Montes S. De Jesus Madrigal M. Burciaga A. Elena Martinez M. Johnson M. J. (2016). Technology-assisted stroke rehabilitation in Mexico: a pilot randomized trial comparing traditional therapy to circuit training in a Robot/technology-assisted therapy gym. J. Neuroeng. Rehabilit. 13, 1–15. 10.1186/s12984-016-0190-1 27634471 PMC5025604

[B10] Calautti C. Baron J. C. (2003). Functional neuroimaging studies of motor recovery after stroke in adults - A review. Stroke 34, 1553–1566. 10.1161/01.STR.0000071761.36075.A6 12738893

[B11] Calautti C. Jones P. S. Guincestre J.-Y. Naccarato M. Sharma N. Day D. J.. (2010). The neural substrates of impaired finger tapping regularity after stroke. Neuroimage 50, 1–6. 10.1016/j.neuroimage.2009.12.012 20004249

[B12] Carey L. M. Abbott D. F. Harvey M. R. Puce A. Seitz R. J. Donnan G. A. (2011). Relationship between touch impairment and brain activation after lesions of subcortical and cortical somatosensory regions. Neurorehabilit. Neur. Rep. 25, 443–457. 10.1177/1545968310395777 21382887

[B13] Chao-Chen L. Lin P. Y. Yu H. Z. Chen J. (2018). “Near infrared spectroscopy study of cortical excitability during electrical stimulation-assisted cycling for neurorehabilitation of stroke patients,” in IEEE Transactions on Neural Systems and Rehabilitation Engineering. p. 1. 10.1109/TNSRE.2018.2829804 29877854

[B14] Cramer S. C. Sur M. Dobkin B. H. O'brien C. Sanger T. D. (2012). Harnessing neuroplasticity for clinical applications. Brain 135, e216–e216. 10.1093/brain/aws018 PMC3102236 21482550

[B15] Duret C. Grosmaire A.-G. Krebs H. I. (2019). Robot-assisted therapy in upper extremity hemiparesis: overview of an evidence-based approach. Front. Neurol. 10, 412. 10.3389/fneur.2019.00412 31068898 PMC6491567

[B16] Han Q. Li Z. Gao Y. Li W. Xin Q. Tan Q.. (2014). Phase synchronization analysis of prefrontal tissue oxyhemoglobin oscillations in elderly subjects with cerebral infarction. Med. Phys. 41. 10.1118/1.4896113 25281981

[B17] Harvey R. L. (2015). Predictors of functional outcome following stroke. Phys. Med. Rehabilit. Clin. 26, 583–598. 10.1016/j.pmr.2015.07.002 26522899

[B18] Horenstein C. Lowe M. J. Koenig K. A. Phillips M. D. (2009). Comparison of unilateral and bilateral complex finger tapping-related activation in premotor and primary motor cortex. Hum. Brain Mapp. 30, 1397–1412. 10.1002/hbm.20610 18537112 PMC6871138

[B19] Hua X. Y. Qiu Y. Q. Wang M. Zheng M. X. Li T. Shen Y. D.. (2016). Enhancement of contralesional motor control promotes locomotor recovery after unilateral brain lesion. Rep 6, 18784. 10.1038/srep18784 26732072 PMC4702126

[B20] Hubbard I. J. Carey L. M. Budd T. W. Levi C. Mcelduff P. Hudson S.. (2015). A randomized controlled trial of the effect of early upper-limb training on stroke recovery and brain activation. Neurorehabil. Neur. Rep. 29, 703–713. 10.1177/1545968314562647 25527488

[B21] Hussar C. R. Pasternak T. (2013). Common rules guide comparisons of speed and direction of motion in the dorsolateral prefrontal cortex. J. Neurosci. 33, 972–986. 10.1523/JNEUROSCI.4075-12.2013 23325236 PMC3711598

[B22] Jakob I. Kollreider A. Germanotta M. Benetti F. Cruciani A. Padua L.. (2018). Robotic and sensor technology for upper limb rehabilitation. PmandR 10, S189–S197. 10.1016/j.pmrj.2018.07.011 30269805

[B23] Kato H. Izumiyama M. Koizumi H. Takahashi A. Itoyama Y. (2002). Near-infrared spectroscopic topography as a tool to monitor motor reorganization after hemiparetic stroke - A comparison with functional MRI. Stroke 33, 2032–2036. 10.1161/01.STR.0000021903.52901.97 12154258

[B24] Koes B. W. Van Tulder M. W. Ostelo R. Burton A. K. Waddell G. (2001). Clinical guidelines for the management of low back pain in primary care - An international comparison. Spine 26, 2504–2513. 10.1097/00007632-200111150-00022 11707719

[B25] Kokotilo K. J. Eng J. J. Boyd L. A. (2009). Reorganization of Brain Function During Force Production After Stroke. J. Neurol. Phys. Ther. 33, 45–54. 10.1097/NPT.0b013e31819824f0 19265770 PMC3186814

[B26] Kokotilo K. J. Eng J. J. Mckeown M. J. Boyd L. A. (2010). Greater Activation of Secondary Motor Areas Is Related to Less Arm Use After Stroke. Neurorehabilit. Neur. Rep. 24, 78–87. 10.1177/1545968309345269 19737873 PMC3181217

[B27] Lindberg P. Schmitz C. Forssberg H. Engardt M. Borg J. (2004). Effects of passive-active movement training on upper limb motor function and cortical activation in chronic patients with stroke: A pilot study. J. Rehabilit. Med. 36, 117–123. 10.1080/16501970410023434 15209454

[B28] Liu N. Cui X. Bryant D. M. Glover G. H. Reiss A. L. (2015). Inferring deep-brain activity from cortical activity using functional near-infrared spectroscopy. Biomed. Opt. Express 6, 1074–1089. 10.1364/BOE.6.001074 25798327 PMC4361422

[B29] Loprinzi P. D. (2019). The effects of exercise on long-term potentiation: a candidate mechanism of the exercise-memory relationship. OBM Neurobiol. 3, 1. 10.21926/obm.neurobiol.1902026

[B30] Lu H. Zuo Y. Gu H. Waltz J. A. Zhan W. Scholl C. A.. (2007). Synchronized delta oscillations correlate with the resting-state functional MRI signal. Proc. Nat. Acad. Sci. 104, 18265–18269. 10.1073/pnas.0705791104 17991778 PMC2084331

[B31] Mehrholz J. Pohl M. Platz T. Kugler J. Elsner B. (2018). Electromechanical and robot-assisted arm training for improving activities of daily living, arm function, and arm muscle strength after stroke. Cochrane Datab. System. Rev. 9, 1–135. 10.1002/14651858.CD006876.pub5 30175845 PMC6513114

[B32] Morone G. Cocchi I. Paolucci S. Iosa M. (2020). Robot-assisted therapy for arm recovery for stroke patients: state of the art and clinical implication. Exp. Rev. Med. Dev. 17, 223–233. 10.1080/17434440.2020.1733408 32107946

[B33] Nee D. E. D'esposito M. (2016). The hierarchical organization of the lateral prefrontal cortex. Elife 5, e12112. 10.7554/eLife.12112.032 26999822 PMC4811776

[B34] Nieuwhof F. Reelick M. F. Maidan I. Mirelman A. Hausdorff J. M. Olde Rikkert M. G. M.. (2016). Measuring prefrontal cortical activity during dual task walking in patients with Parkinson's disease: feasibility of using a new portable fNIRS device. Pilot Feasibil. Stud. 2, 59–59. 10.1186/s40814-016-0099-2 27965875 PMC5154104

[B35] Pan L. Song A. Xu G. (2012). “Robot-assisted upper-limb fuzzy adaptive passive movement training and clinical experiment”, in 3rd International Conference on Mechanical and Electronics Engineering (ICMEE 2011). p. 227. 10.4028/www.scientific.net/AMM.130-134.227

[B36] Pascualleone A. Amedi A. Fregni F. Merabet L. B. (2005). The plastic human brain cortex. Ann. Rev. Neurosci. 28, 377. 10.1146/annurev.neuro.27.070203.144216 16022601

[B37] Patrice V. Thomas M. E. Miguel C. F. J. tienne D.V.-S. (2017). Dynamic brains and the changing rules of neuroplasticity: implications for learning and recovery. Front. Psychol. 8, 1657. 10.3389/fpsyg.2017.01657 29085312 PMC5649212

[B38] Pekna M. Pekny M. Nilsson M. (2012). Modulation of neural plasticity as a basis for stroke rehabilitation. Stroke 43, 2819–2828. 10.1161/STROKEAHA.112.654228 22923444

[B39] Pfeifer M. Felix S. Rob L. (2017). Signal processing in functional near-infrared spectroscopy (fnirs): methodological differences lead to different statistical results. Front. Hum. Neurosci. 11, 641. 10.3389/fnhum.2017.00641 29358912 PMC5766679

[B40] Rensink M. Schuurmans M. Lindeman E. Hafsteinsdottir T. (2009). Task-oriented training in rehabilitation after stroke: systematic review. J. Adv. Nurs. 65, 737–754. 10.1111/j.1365-2648.2008.04925.x 19228241

[B41] Riecker A. Gröschel K. Ackermann H. Schnaudigel S. Kassubek J. Kastrup A. (2010). The role of the unaffected hemisphere in motor recovery after stroke. Hum. Brain Mapp. 31, 1017–1029. 10.1002/hbm.20914 20091792 PMC6870650

[B42] Rodgers H. Bosomworth H. Krebs H. I. Van Wijck F. Howel D. Wilson N.. (2019). Robot assisted training for the upper limb after stroke (RATULS): a multicentre randomised controlled trial. Lancet 394, 51–62. 10.1016/S0140-6736(19)31055-4 31128926 PMC6620612

[B43] Santosa H. Hong M. J. Kim S.-P. Hong K.-S. (2013). Noise reduction in functional near-infrared spectroscopy signals by independent component analysis. Rev. Scient. Instrum. 84, 073106. 10.1063/1.4812785 23902043

[B44] Scholkmann F. Kleiser S. Metz A. J. Zimmermann R. Wolf M. (2014). A review on continuous wave functional near-infrared spectroscopy and imaging instrumentation and methodology. Neuroimage 85, 6–27. 10.1016/j.neuroimage.2013.05.004 23684868

[B45] Smith G. S. (2013). Aging and neuroplasticity. Dial. Clin. Neurosci. 15, 3. 10.31887/DCNS.2013.15.1/gsmith PMC3622467 23576885

[B46] Tachtsidis I. Elwell C. E. Leung T. S. Lee C. W. Delpy D. T. (2004). Investigation of cerebral haemodynamics by near-infrared spectroscopy in young healthy volunteers reveals posture-dependent spontaneous oscillations. Physiol. Measurem. 25, 437–445. 10.1088/0967-3334/25/2/003 15132309

[B47] Tan Q. Zhang M. Wang Y. Zhang M. Wang B. Xin Q.. (2016). Age-related alterations in phase synchronization of oxyhemoglobin concentration changes in prefrontal tissues as measured by near-infrared spectroscopy signals. Microv. Res. 103, 19–25. 10.1016/j.mvr.2015.10.002 26525098

[B48] Tan Q. Zhang M. Wang Y. Zhang M. Wang Y. Xin Q.. (2015). Frequency-specific functional connectivity revealed by wavelet-based coherence analysis in elderly subjects with cerebral infarction using NIRS method. Med. Phys. 42, 5391–5403. 10.1118/1.4928672 26328988

[B49] Waddell K. J. Birkenmeier R. L. Bland M. D. Lang C. E. (2016). An exploratory analysis of the self-reported goals of individuals with chronic upper-extremity paresis following stroke. Disabil. Rehabilit. 38, 853–857. 10.3109/09638288.2015.1062926 26146964 PMC4809414

[B50] Willie C. K. Tzeng Y.-C. Fisher J. A. Ainslie P. N. (2014). Integrative regulation of human brain blood flow. J. Physiol. 592, 841–859. 10.1113/jphysiol.2013.268953 24396059 PMC3948549

[B51] Wu Q. Yue Z. Ge Y. Ma D. Yin H. Zhao H.. (2020). Brain functional networks study of subacute stroke patients with upper limb dysfunction after comprehensive rehabilitation including BCI training. Front. Neurol. 10, 1419. 10.3389/fneur.2019.01419 32082238 PMC7000923

[B52] Xie H. Zhang M. Huo C. Xu G. Li Z. Fan Y. (2019). Tai Chi Chuan exercise related change in brain function as assessed by functional near-infrared spectroscopy. Scient. Rep. 9, 1–14. 10.1038/s41598-019-49401-9 31519933 PMC6744459

[B53] Xu L. Wang B. Xu G. Wang W. Liu Z. Li Z. (2017). Functional connectivity analysis using fNIRS in healthy subjects during prolonged simulated driving. Neurosci. Lett. 640, 21–28. 10.1016/j.neulet.2017.01.018 28087436

[B54] Zhang H. Zhang Y.-J. Lu C.-M. Ma S.-Y. Zang Y.-F. Zhu C.-Z. (2010). Functional connectivity as revealed by independent component analysis of resting-state fNIRS measurements. Neuroimage 51, 1150–1161. 10.1016/j.neuroimage.2010.02.080 20211741

